# Supplementary material for: Off-Target Effect of Activation of NF-κB by HIV Latency Reversal Agents on Transposable Elements Expression
Source: Viruses. 2022 Jul 20;14(7):1571. doi: 10.3390/v14071571 (PMC9318874; doi:10.3390/v14071571)
Supplement: Supplementary file 1 [file viruses-14-01571-s001.zip › viruses-1768737-supplementary.pdf]

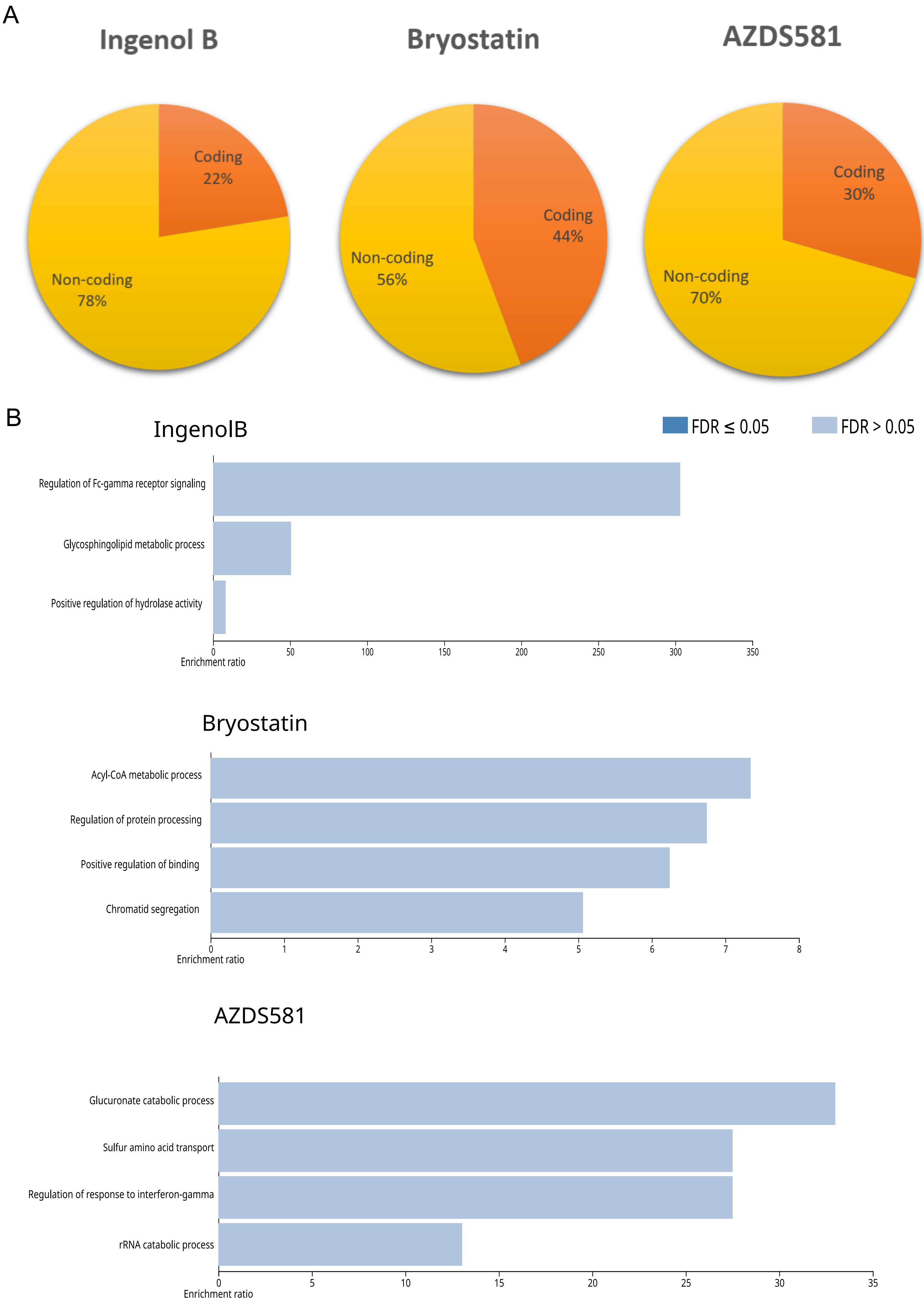

Figure S1. Proportion of coding and noncoding differentially expressed retro elements (**A**) and en-riched neighboring gene analysis (**B**) for each type of treatment.
